# Scanning Electron Microscopy-Based Approach to Understand the Mechanism Underlying the Adhesion of Dengue Viruses on Ceramic Hydroxyapatite Columns

**DOI:** 10.1371/journal.pone.0053893

**Published:** 2013-01-10

**Authors:** Maiko Saito, Yae Kurosawa, Tsuneo Okuyama

**Affiliations:** 1 R&D Department, PENTAX New Ceramics Division, HOYA Corporation, Tokyo, Japan; 2 Protein Technos Institute, Kanagawa, Japan; University of South Florida College of Medicine, United States of America

## Abstract

Although ceramic hydroxyapatite (HAp) chromatography has been used as an alternative method ultracentrifugation for the production of vaccines, the mechanism of virus separation is still obscure. In order to begin to understand the mechanisms of virus separation, HAp surfaces were observed by scanning electron microscopy after chromatography with dengue viruses. When these processes were performed without elution and with a 10–207 mM sodium phosphate buffer gradient elution, dengue viruses that were adsorbed to HAp were disproportionately located in the columns. However, when eluted with a 10–600 mM sodium phosphate buffer gradient, few viruses were observed on the HAp surface. After incubating the dengue viruses that were adsorbed on HAp beads at 37°C and 2°C, the sphericity of the dengue viruses were reduced with an increase in incubation temperature. These results suggested that dengue virus was adsorbed to the HAp surface by electronic interactions and could be eluted by high-salt concentration buffers, which are commonly used in protein purification. Furthermore, virus fusion was thought to occur with increasing temperature, which implied that virus-HAp adhesion was similar to virus-cell adhesion.

## Introduction

The methodology of density-gradient ultracentrifugation, which has traditionally been applied in the purification of viruses, is able to achieve satisfactory results in vaccine production. However, the ultracentrifugation process is cumbersome and time-consuming [1]. In recent years, several types of chromatographic procedures have been applied as alternative methods to ultracentrifugation, and many studies that have used these procedures have been published [2], [3], [4]. Hydroxyapatite (HAp) chromatography is another substitute methodology for ultracentrifugation. HAp has the property of adsorbing massive organic particles, such as cells and viruses [5], [6]. Thus, HAp chromatography is thought to enable a variety of viruses to be purified. Furthermore, HAp has achieved satisfactory results in large-scale protein purifications. Therefore, virus purification by HAp chromatography might be suitable for industry usage. The mechanisms underlying virus adsorption to HAp are still obscure. However, reports that have demonstrated favorable isolations of viruses have been published previously, and good results were obtained with high-salt eluting as well as with protein isolation [7], [8], [9].

In this study, in order to visually reveal some of the mechanisms underlying virus purification by HAp chromatography, we attempted to observe the location of adsorbed viruses with a scanning electron microscope (SEM) and count the number of dengue viruses that were adsorbed onto the HAp surface. Although viruses in column effluent have been analyzed previously, this study offers a new perspective that would help to understand viral behaviors inside a chromatographic column.

Furthermore, the dengue virus morphologies on the HAp surface after the incubation processes were observed by SEM in order to understand the virus-HAp interactions. The dengue virus is a member of the flavivirus family that carries a single-stranded RNA with an envelope and has a virus diameter of approximately 50–60 nm [6]. Although it has been estimated that 50 million dengue virus infections occur globally each year, the development of an effective dengue vaccine has not been achieved [10].

## Materials and Methods

### 1. Virus and Cell

Dengue virus strain type 2 ThNH7/93 and *Aedes albopictus mosquito* C6/36 cells [11] were provided by Professor K. Morita of the Department of Virology, Institute of Tropical Medicine, Nagasaki University, Japan.

### 2. Cell Culture and Virus Production

The cell culture medium used was Eagle’s Minimum Essential Medium with l-glutamine (MP Biomedicals LLC, Solon, OH, USA) that was supplemented with 10% fetal calf serum.

The virus production medium used was Autopow- Eagle’s Minimum Essential Medium without l-glutamine (MP Biomedicals LLC) supplemented with 0.5% fetal calf serum.

C6/36 cells were grown in 45 mL of the cell culture medium in a 225-cm^2^ culture flask that was precoated with 100 µg/mL of poly-l-lysine (Sigma-Aldrich Co., St. Louis, MO, USA) at 28°C for 7 days in order to reach confluence. The dengue virus was then inoculated onto the cells in 75 mL of the virus production medium. After culturing for 3 days, the medium was changed with 75 mL of fresh medium and then cultured at 28°C for a further 4 days (total culture period was 7 days). We subsequently collected 75 mL of the 7-day culture fluid. The dengue virus culture fluid was filtered through a 0.22-µm pore-size membrane.

### 3. Preparation of HAp Columns

HAp suspension (Ca/P = 1.67) was prepared using calcium hydroxide and phosphoric acid. The suspension was dried to obtain spherical particles (average diameter, 40 µm) by using a spray-dry technique, and then, the particles were sintered at 950°C. The particles were packed into an empty stainless-steel column (4.0 I.D.×35 mm; Sugiyama Shoji Co., Ltd., Kanagawa, Japan). Three columns (represented as Column A, Column B, and Column C) were prepared for use with 3 different elution processes, as described in the Materials and methods, section 5. Another 3 columns (represented as Column D, Column E, and Column F) were prepared for incubation under 3 different conditions, as described in the Materials and methods, section 6.

### 4. Virus Adsorption to HAp Columns in the Chromatography System

Dengue virus adsorption to Columns A–F was performed using the BioLogic chromatography system (Bio-Rad Laboratories, Inc., Hercules, CA, USA). Virus culture fluid (25 mL) was loaded onto each column for adsorption, and the columns were equilibrated with 45 column volumes (CV) of 10 mM sodium phosphate buffer (NaPB), pH 7.2. The flow rate was adjusted throughout at 1 mL/min. The effluents were monitored at 280 nm and 260 nm. The fractions were collected every minute.

### 5. Virus Elution Using the Chromatography System

Dengue viruses were similarly adsorbed to Columns A–C, as described in the Materials and methods, section 4, as follows: Column A, no elution was performed; Column B was eluted with a 10–207 mM NaPB (pH 7.2) gradient (11 CV); and Column C was eluted with a 10–600 mM NaPB (pH 7.2) gradient (34 CV). The flow rate, monitoring of the effluents, and collection of the fractions were conducted under the same conditions as described in the Materials and methods, section 4. There were no time lags between the virus adsorption processes described in the Materials and methods, section 4, and the elution processes described in the Materials and methods, section 5.

### 6. Incubation of the Dengue Viruses that were Adsorbed onto HAp Media at 2°C or 37°C

Dengue viruses were similarly adsorbed onto Columns D–F, as described in the Materials and methods, section 4. Column D was not incubated, Column E was incubated at 2°C for 7 days, and Column F was incubated at 37°C for 7 days.

### 7. Pretreatment of HAp Particles and Observation by SEM

**Figure 1:**
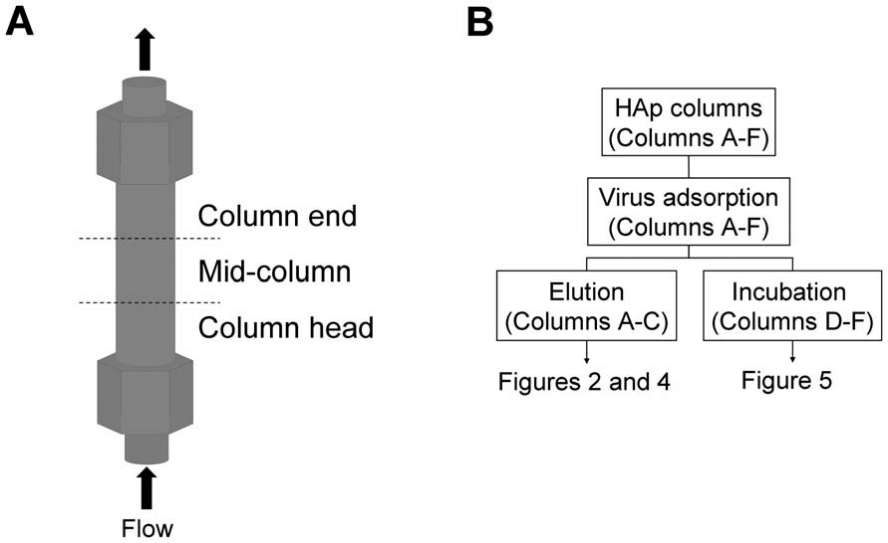
Illustration of experimental methods. A: The flow direction and the division of the hydroxyapatite (HAp) column into 3 parts. After removing the HAp particles from the column, HAp particles were collected in each part of the column. Column head: lower position of the column (upstream side); Mid-column: middle position of the column; Column end: upper position of the column (downstream side). B: Virus elution and incubation processes of HAp columns. Detailed conditions are described in the Materials and methods, section 3–6.

After each process, Columns A–F were unpacked by pushing the column out with a syringe while being careful not to mix the HAp particles of the different parts of the column. HAp particles that were unpacked from Columns A–C were collected from the 3 sections of each column: the column head, the mid-column, and the column end (Figure 1A).

However, HAp particles that were unpacked from Columns D–F were collected only from the column head. Each collected particle was fixed with 1% glutaraldehyde (Wako Pure Chemical Industries, Ltd., Osaka, Japan) in 100 mM NaPB (pH 7.4), for 30 min and washed 3 times in 100 mM NaPB (pH 7.4). The particles were further fixed with 1% osmium (VIII) oxide (Wako Pure Chemical Industries, Ltd.) in 100 mM NaPB (pH 7.4), for 30 min and washed 3 times in 10 mM NaPB (pH 7.4).

Each HAp particle in 10 mM NaPB (pH 7.4), was mounted onto a metal plate with carbon tape and dried at room temperature. The particles were coated with platinum-palladium in an E-1030 Ion Sputter (Hitachi High-Technologies Corporation, Tokyo, Japan) and observed at 8.0 kV or 10 kV with an S-4200 or S-4300 scanning electron microscope (Hitachi High-Technologies Corporation). The process flow chart of the elutions and incubations, as described in the Materials and methods, sections 3–7, are shown in Figure 1B.

The viruses adsorbed on HAp were identified as dengue viruses if they were of a particle diameter within the range 40–60 nm and if their shape was spherical. The sphericity of the virus particle was determined by calculating the difference between 2 orthogonal diameter measurements; if this difference was less than 20%, the viruses were classified as spherical, and if the difference was more than 20%, they were classified as flat.

### 8. Hemagglutination (HA) Test

Goose red blood cells (Nippon Biotest Laboratories Inc., Tokyo, Japan) were washed with phosphate-buffered saline (PBS) and suspended in PBS at a final concentration of 8% as a stock solution. This stock solution was diluted to 1∶24 in order to prepare a 0.33% goose red blood cell working suspension in virus-adjusting diluent containing 150 mM sodium chloride and 200 mM NaPB with different pH values (pH 6.2, 6.4, and 6.6). The optimal pH was selected to show the highest titer among them. The virus sample (50 µL) was diluted 2-fold serially with 0.4% bovine serum albumin in BS9 (120 mM sodium chloride, 50 mM boric acid, and 24 mM sodium hydrate). Fifty microliters of the 0.33% red blood cell suspension was then added to each well, the plate was mixed gently, and it was incubated at 37°C for 30 min. The HA titer that was the highest dilution of virus showed the agglutination pattern on the bottom of the well.

## Results

### HAp Chromatography and HA Test

**Figure 2:**
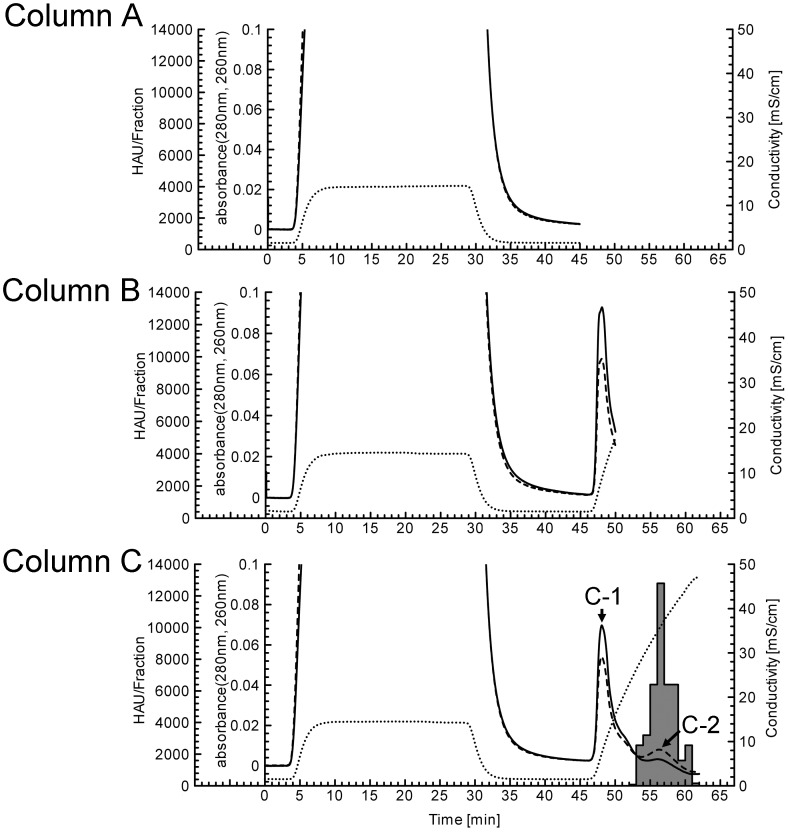
HAp chromatogram of dengue virus culture fluids from 3 different elution processes. The solid line is the UV absorbance at 280 nm, the broken line is the UV absorbance at 260 nm, and the dashed line is the conductivity of the elution. The shaded histogram indicates the hemagglutination unit of each fraction. Column A: No elution; Column B: 10–207 mM NaPB (pH 7.2) gradient elution; Column C: 10–600 mM NaPB (pH 7.2) gradient elution. C-1: Admixture proteins peak; C-2: Virus peak.

Dengue viruses were adsorbed to HAp particles in Columns A–C, and a different elution process was performed in each column. As shown in Figure 2, for Columns A–C, broad UV peaks at 280 nm and peaks at 260 nm, which were thought to be the sample loading peak, were detected from 4 min to 35 min. However, HA units were not detected in any of the fractions of the loading peaks of Columns A–C. For Column B, one UV peak at 280 nm and one peak at 260 nm, other than the sample-loading peak, were detected with the initial elution gradient. However, HA units were not detected in any of the fractions of Column B (Figure 2). For Column C, 2 UV peaks at 280 nm and at 260 nm, other than the sample-loading peak, were detected. The first of the 2 UV peaks (C-1) was detected with the initial elution gradient, as in Column B, and the last peak (C-2) was detected behind the C-1 UV peak. HA units were detected from 55 min to 65 min, which corresponded to the retention time of the C-2 UV peak. Furthermore, the C-2 UV peak at 260 nm was a higher intensity than that at 280 nm, and this was thought to reflect the fact that the dengue virus is a single-stranded RNA. The results showing that HA units were not detected in the fractions of the C-1 UV peak suggested that an admixture of proteins was eluted in the C-1 UV peak. Therefore, Figure 2 shows that dengue viruses were not eluted from Column A and B but were eluted from Column C with a later gradient.

### Observations of Adsorbed Viruses in the HAp Columns

**Figure 3:**
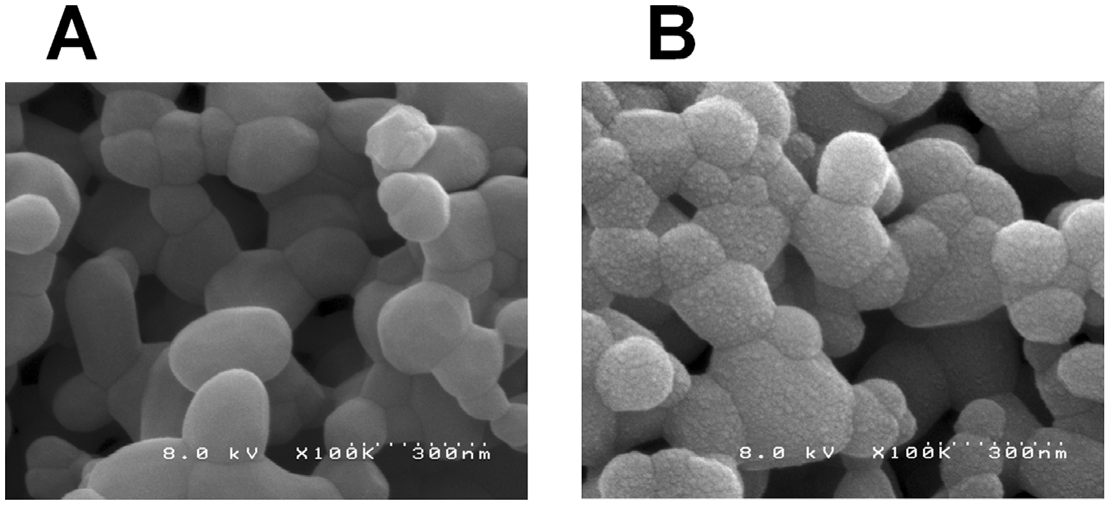
SEM images of control HAp surfaces. A: Untreated HAp particles; B: HAp particles after loading of cell culture fluid (virus-free).

**Figure 4:**
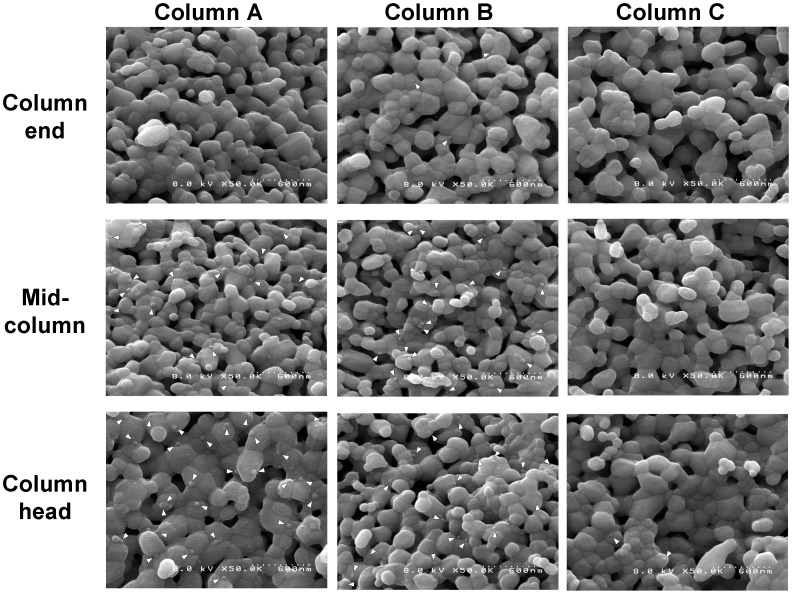
Observation of HAp surfaces that were used in the 3 different elution processes. Column A: No elution; Column B: 10–207 mM NaPB (pH 7.2) gradient elution; Column C: 10–600 mM NaPB (pH 7.2) gradient elution. Each column was divided into 3 sections (Column end, Mid-column, Column head). The white triangles indicate dengue viruses.

Figure 3 shows a comparison between untreated HAp particles that were not loaded with either virus-free or the virus samples (Figure 3A) and untreated HAp particles after loading cell culture fluid (virus-free) (Figure 3B). Untreated HAp particles and HAp particles after loading of cell culture fluid (virus-free) were prepared via the methods described in the Materials and methods, sections 3–7. Three different elution processes were performed on Columns A–C. The HAp particles were collected from the 3 parts (column head, mid-column, and column end) of each column (Figure 1A.), and the collected HAp particles were observed by SEM (Figure 4). Furthermore, the virus count was determined, as shown in Figure 4; the count was repeated, and the average of the counts is indicated in Table 1. For the Column A condition where no elution was performed, dengue viruses on the HAp surfaces of the column head and mid-column sections were visually confirmed. However, no viruses were observed on the HAp surface of the column end. Therefore, most of the loaded dengue viruses were disproportionately adsorbed onto the HAp surface of the inlet side of the column. In addition, statistical analysis showed a significant difference between the inlet side and the middle portion of the column (column end vs. mid-column; P<.05, by Student’s *t* test).

**Table 1:** The average number of dengue viruses that were observed in the SEM images.

	Column A	Column B	Column C
Column end	0 (±0)	2 (±1)	0 (±0)
Mid-column	16 (±5)	15 (±4)	0 (±0)
Column head	30 (±10)	13 (±5)	2 (±1)

Standard deviation in brackets.

For the Column B condition (elution with a 10–207 mM NaPB gradient), the numbers of adsorbed dengue viruses on the HAp surface of the column head and mid-column sections were nearly equivalent. However, the number of dengue viruses that were adsorbed onto the HAp surface of the column end section was quite small. We assumed that, at the beginning of the NaPB gradient, some viruses were desorbed by the low NaPB, and the viruses then moved to the outlet side of the column. Eventually, the drifting viruses were again adsorbed to downstream HAp particles. Statistical analysis also showed a significant difference between the column heads of Column A and Column B (Column A vs. Column B; P<.05, by Student’s *t* test), indicating the occurrence of a virus drift.

For the Column C condition (elution with a 10–600 mM NaPB gradient), only 2 dengue viruses were observed on the HAp surface of the column head section, and no dengue viruses were observed on the HAp surface of the column end and mid-column sections. As shown in Figure 4 and Table 1, the number of dengue viruses that were adsorbed onto the HAp surface of the column head was decreased, and the numbers of viruses that were adsorbed onto the mid-column and column end increased with increasing NaPB concentration. However, almost all of the viruses that were adsorbed were desorbed from the HAp surface by the 600-mM NaPB gradient elution. It was clear that dengue viruses could not be adsorbed to HAp in a high concentration of NaPB and were ejected from the column.

Accordingly, the following findings of virus behaviors are visualized in Figure 4: (1) many dengue viruses were disproportionately adsorbed onto the HAp of the column head side before NaPB elution, (2) viruses that drifted to the column end were desorbed and readsorbed to the HAp surface repeatedly in the halfway gradient elution, and (3) viruses became less absorbable to the HAp surface with a high concentration of NaPB. The behaviors listed above are similar to the fundamental principles of protein separation with adsorption chromatography, suggesting that the behaviors of dengue viruses passing through the HAp column were nearly analogous to that of proteins [12].

### Alteration of Virus Shape by Temperature Change

**Figure 5:**
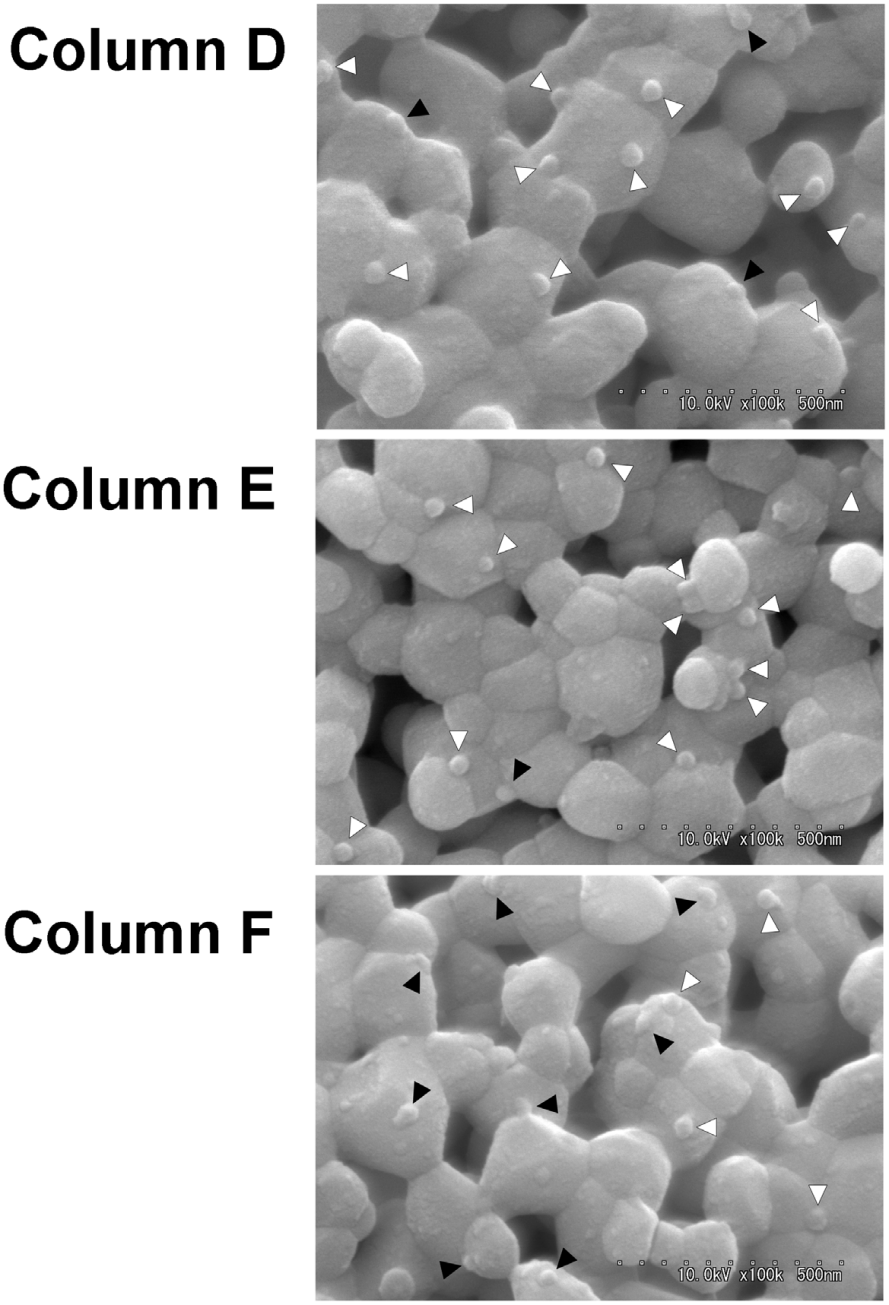
Observation of HAp surfaces that were incubated under 3 different conditions. Column D: no incubation; Column E: incubated at 2°C for 7 days; Column F: incubated at 37°C for 7 days. HAp particles of the column head were observed with scanning electron microscopy. The white triangles indicate spherical-shaped viruses. The black triangles indicate flat-shaped viruses.

Figure 5 shows that the alterations in the dengue virus shapes that occurred on HAp surfaces due to temperature were visible. The virus count was determined as shown in Figure 5, and the shapes of the dengue viruses adhered to HAp were classified as either spherical or flat on the basis of virus sphericity. The counts were repeated and the average is indicated in Table 2. For Column D (not incubated), the percentage of viruses with a spherical shape was 67% (8/12). For Column E (incubated at 2°C for 7 days) and Column F (incubated at 37°C for 7 days), the percentages of viruses with a spherical shape were 83% (10/12) and 45% (5/11), respectively. Most viruses that were incubated at 2°C (Column E) remained adhered to the HAp surface with better sphericity. In contrast, many of the viruses that were incubated at 37°C (Column F) had flat shapes, and the number of spherically shaped viruses was decreased compared to those in Column D. Given that the viruses on Column D were subjected to the whole process at room temperature, the numbers of spherically shaped viruses observed were likely to increase in the order of Column E, Column D, and Column F. Student’s *t* test analysis also showed significant differences between Column E and Column F (P<.01), between Column E and Column D (P<.01), and between Column D and Column F (P<.1). The shapes of the dengue viruses that were adsorbed to the HAp surface were altered from spherical to flat shapes with increasing temperature.

**Table 2:** The average number of dengue viruses that were observed and counted separately as spherical viruses and flat viruses on the SEM images.

	Spherical shape	Flat shape
Column D	8 (±2)	4 (±2)
Column E	10 (±2)	2 (±1)
Column F	5 (±2)	6 (±2)

Standard deviation in brackets.

## Discussion

The HAp chromatograms showed that the dengue viruses were separable from admixture proteins with HAp chromatography as well as with commonly used protein purification methods. However, concerning the width of the peaks, the C-1 UV peak for eluting admixture proteins was narrower than the C-2 UV peak for eluting dengue viruses (Figure 2). The dengue virus diameter is approximately 50 nm. Virus particles are quite large compared to proteins, and virus diffusion is 2 to 100-fold slower than that of proteins [2]. The migration of viruses through the void between HAp particles is also slower than that of proteins, and, hence, the C-2 UV peak appeared to be broad.

The SEM images showed the in-column circumstances of the viruses on the HAp surface of the NaPB gradient from the start to the end (Figure 4). The debris and impurities on the HAp particles after loading of virus samples (Figures 4 and 5) were slightly larger than those on the HAp particles after loading of virus-free sample (Figure 3B). These debris and impurities may have accumulated before the virus sample was adsorbed and incubated.

When comparing the chromatogram and SEM images, when HA units were not detected in any of the fractions, viruses could be observed in the corresponding SEM images. The opposite is shown in Figures 2 and 4. The consequence of HA units in the fractions and the observation of viruses in the corresponding SEM images visually confirmed that dengue viruses could be eluted by high-salt concentrations with HAp chromatography, which is consistent with the results reported by other researchers [7], [8], [9]. HAp possesses cation-exchange capabilities and calcium metal affinities, which contribute to the elution of dengue viruses. Therefore, the adhesion of dengue viruses to HAp could be regarded as occurring through adsorption by electronic interactions, as well as through that of protein-HAp interactions. The similarity in the interactions between virus-HAp and protein-HAp implies that surface proteins of the virus particles are important for adhesion, indicating that the adhesion mechanism would depend on the surface charge of the proteins that constitute the virus particles. We have already identified that during elution, the duration for which the other serotypes of the dengue virus were retained on the HAp column was different from the retention time for type 2 (data not shown). Additionally, many reports of virus elutions by high-salt concentrations with ion exchange chromatography have been published, and their results have implied that virus-resin adsorptions are also predicted to be similar to the protein elution mechanisms [13], [14], [15], [16].

Figure 5 shows that the shapes of the dengue viruses on the HAp surfaces changed to flat and nonspherical with increasing temperatures. As is well known, virus-cell binding occurs via various receptors [17]. However, the mechanisms underlying how receptors bind to the envelope proteins of dengue viruses remain unclear [18]. In addition, whether endocytosis or direct fusion is involved in the cell-entry mechanisms of dengue viruses has been a controversial subject [19]. In this study, the virus-HAp complex appeared to be bound by electronic interactions between HAp and the envelope protein of the dengue virus because HAp does not have receptors and the elution behavior of dengue viruses with HAp chromatography is similar to that of proteins. Nevertheless, because virus morphology and virus-HAp adhesion were altered by rising temperatures, it was presumed that the envelope fusion that occurred was not receptor mediated. Virus fusion has been shown to progress with liposomes depending on temperature in past reports. For instance, Gollins and Porterfield reported that fusion between the West Nile virus and liposomes occurred rapidly at low pH and high temperature [20], [21]. In this study, dengue viruses were bound to the HAp surface by electronic interactions, and, subsequently, virus envelope fusion was thought to occur with temperature dependence. These results suggested that virus-HAp interactions were substantially similar to virus-cell interactions. Furthermore, the electronic interactions between dengue viruses and cells might be acting in the role of cell entry and infection of dengue viruses. HAp has been used as a biocompatible material for many years, and its biological potential has been distinguished from other chemical synthetics. Thus, virus morphological alterations were assumed [22].

### Conclusion

Dengue virus infections have increased in recent decades. Thus, vaccine development has also been active [23]. Purification by ultracentrifugation has traditionally played a leading role in vaccine production. However, chromatography, including that of ceramic HAp, has recently become an alternative method. Thus, obtaining reliable and robust purifications by chromatography is one of the priority issues in vaccine production. This study showed that the mechanisms of adsorption of viruses on-HAp are similar to the general mechanisms of protein elution, and that HAp has a strong affinity for dengue viruses. Therefore, in addition to its common application in protein purification, HAp chromatography could be effective for virus purification. Visualization of the ceramic HAp chromatography process of the dengue virus could assist in establishing virus purification procedures for the laboratory and industry and reveal the interactions between dengue viruses and HAp and/or cells.
